# RNAmotifs: prediction of multivalent RNA motifs that control alternative splicing

**DOI:** 10.1186/gb-2014-15-1-r20

**Published:** 2014-01-31

**Authors:** Matteo Cereda, Uberto Pozzoli, Gregor Rot, Peter Juvan, Anthony Schweitzer, Tyson Clark, Jernej Ule

**Affiliations:** 1 Scientific Institute IRCCS E. Medea, Via don L. Monza 20, 23842 Bosisio Parini, (LC), Italy; 2 MRC Laboratory of Molecular Biology, Hills Road, Cambridge CB2 0QH, UK; 3 Department of Experimental Oncology, European Institute of Oncology (IEO), 20139 Milan, Italy; 4 Institute of Molecular Life Sciences and Swiss Institute of Bioinformatics, Winterthurerstrasse 190, CH-8057 Zurich, Switzerland; 5 Faculty of Medicine, University of Ljubljana, Vrazov trg 2, SI-1104 Ljubljana, Slovenia; 6 Faculty of Mathematics, Natural Sciences and Information Technologies, University of Primorska, Glagoljaška 8, SI-6000 Koper, Slovenia; 7 Affymetrix, 3420 Central Expressway, Santa Clara, CA 95051, USA; 8 Department of Molecular Neuroscience, UCL Institute of Neurology, Queen Square, London WC1N 3BG, UK

## Abstract

RNA-binding proteins (RBPs) regulate splicing according to position-dependent principles, which can be exploited for analysis of regulatory motifs. Here we present RNAmotifs, a method that evaluates the sequence around differentially regulated alternative exons to identify clusters of short and degenerate sequences, referred to as multivalent RNA motifs. We show that diverse RBPs share basic positional principles, but differ in their propensity to enhance or repress exon inclusion. We assess exons differentially spliced between brain and heart, identifying known and new regulatory motifs, and predict the expression pattern of RBPs that bind these motifs. RNAmotifs is available at https://bitbucket.org/rogrro/rna_motifs.

## Background

The majority of human genes produce multiple mRNA isoforms via the process of alternative splicing [1]. Alternative splicing is regulated mainly by RNA-binding proteins (RBPs), which often act according to positional principles defined by an RNA splicing map to enhance or repress exon inclusion [2,3]. These RBPs play key roles in development and evolution, and mutations perturbing protein-RNA interactions can lead to a variety of diseases [4,5]. Therefore, to infer the splicing regulatory programs and identify new disease-causing mutations, algorithms are required that can assess the genomic sequence at the differentially regulated exons to predict the RNA motifs bound by these RBPs.

Great progress has been made over the past decade in inferring the programs of splicing regulation [1]. However, it is not yet clear which positional principles of splicing regulation are shared between different RBPs. The sites of protein-RNA interactions have been defined by different crosslinking and immunoprecipitation (CLIP) methods (HITS-CLIP, PAR-CLIP or iCLIP), but the differences between these methods preclude precise comparisons between the RNA maps that were derived for the different RBPs [3]. Moreover, crosslinking-based methods are affected by mild sequence biases [6]; thus, it is important to develop methods that can derive the regulatory motifs independently of the CLIP data. Therefore, a new computational method is required to derive RNA maps solely from the analysis of gene expression data.

Past studies that predicted splicing regulatory motifs from analysis of the differentially regulated exons searched for continuous motifs, which most often identified UGCAUG as the most frequent motif [7-15]. This sequence is recognized by RNA binding protein, fox-1 homologs 1 and 2 (RBFOX1 and RBFOX2), splicing regulators that recognize three nucleotides via the canonical RNA binding surface and an additional four nucleotides via the loops of a quasi-RRM (qRRM) domain [16]. However, RBFOX proteins are exceptional in their ability to recognize a long continuous motif, and most other splicing regulators recognize motifs that are only three or four nucleotides long [17,18].

Studies of neuro-oncological ventral antigen 1 and 2 (NOVA1 and NOVA2), here collectively referred to as NOVA proteins, demonstrated that three or more short RNA motifs that are clustered closely together on the pre-mRNA are required for NOVA proteins to mediate splicing regulation [2]. Here we will refer to these motifs as 'multivalent RNA motifs', since they enable RBPs to achieve high-affinity binding by cooperative interactions between multiple RNA-binding domains and the clustered short RNA motifs [17,18]. Past computational methods for analysis of multivalent RNA motifs have focused on the known RNA motifs [19], or have predicted motifs based on the CLIP studies of protein-RNA interactions [17,18]. However, a method for *de novo* identification of multivalent RNA motifs by analysis of the regulated exons is not yet available.

Here, we present RNAmotifs, a method that identifies clusters of short non-degenerate (ND) or degenerate (DG) tetramers that are enriched at specific positions around the enhanced and silenced exons. The method correctly identified the multivalent RNA motifs bound by NOVA, PTBP1, heterogeneous nuclear ribonucleoprotein C (hnRNP C), TARDBP, and TIA1 and TIAL1 cytotoxic granule-associated RNA binding proteins (here collectively referred to as TIA proteins). Moreover, RNAmotifs determines the RNA splicing map, which enabled us to compare the positional principles of different RBPs. Finally, we analyzed the exons that are differentially spliced between brain and heart, identifying new candidate motifs responsible for tissue-specific splicing regulation. Notably, we demonstrate that the positional enrichment information of the RNA splicing map can be used to predict the tissue where the candidate regulatory protein that binds each RNA motif is more highly expressed.

## Results

### Identification of multivalent regulatory motifs

In recent years, exon and splice junction microarray and RNAseq studies have identified groups of exons that are differentially regulated in specific tissues and diseases or at specific developmental stages [1]. In spite of the abundance of expression data, it remains challenging to identify the transacting factors that control splicing of the differentially regulated exons. Here we exploited the clustering property of regulatory motifs to develop RNAmotifs, a method for *de novo* identification of multivalent regulatory motifs. We considered tetramers as the core motifs, assuming that most RNA-binding domains recognize up to four nucleotides [17]. We evaluated 64 DG tetramers, where purine R(A/G) or pyrimidine Y(C/T) transitions were allowed at the boundary nucleotides, such as in the YCAY tetramer. The degeneracy was allowed because several RBPs tolerate purine or pyrimidine transitions in their target motifs [17,20-23]. To identify multivalent motifs, we assessed if the motifs were clustered with spacing of up to 15 nucleotides, which we chose based on previous studies of PTBP1 and NOVA motif spacing [21,24].

**Figure 1:**
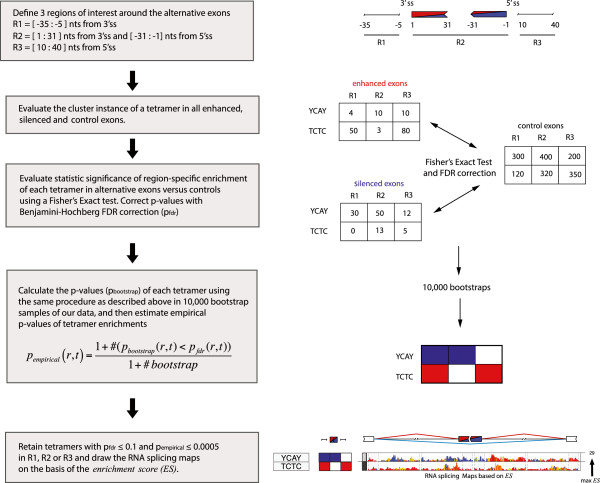
**Flowchart describing the steps used by RNAmotifs to identify the enriched multivalent RNA motifs.** The multivalent RNA motifs are predicted by assessing clusters of tetramers that are enriched in the genomic sequence at specific positions relative to enhanced or silenced exons, compared to control exons. Clusters of each tetramer are evaluated in three regions around the splice sites of alternative exons. Analysis of control exons is used to determine the clustering threshold that each tetramer needs to reach before it is considered as a ‘cluster instance’. A one-tailed Fisher’s exact test is then used to test the null hypothesis that the number of cluster instances at a precise region of a particular tetramer is not different between enhanced (or silenced) and control exons, and the Benjamini-Hochberg false discovery rate (FDR) correction is applied to calculate p_*fdr*_. For each tetramer, the achieved significance level of the test (p_empirical_) is calculated with a bootstrap procedure using 10,000 samples. Tetramers with p_*fdr*_ ≤0.1 and p_empirical_ ≤0.0005 in at least one region either in the enhanced or silenced set are retained (Additional file 2). The RNA map is then drawn to visualize the enrichment score at each nucleotide around the enhanced or silenced exons, and their flanking exons. nts, nucleotides; ss, splice site.

We evaluated the genomic sequence at three regions around the splice sites of the regulated exons (Figure 1). These regions were defined based on the RNA splicing map of NOVA proteins, which has been determined by the positioning of conserved YCAY clusters as well as by the binding sites identified by HITS-CLIP [2,14,25]. We analyzed tetramer clusters in these regions by evaluating enrichment in enhanced and silenced exons, compared to control exons. Each region in enhanced and silenced exons was evaluated separately, because RBPs generally bind at different positions when they enhance or silence exon inclusion [3]. We determined region-specific enrichment of each motif using Fisher’s exact test, corrected this for multiple testing, and calculated the achieved significance level of the test using a bootstrapping procedure.

**Figure 2:**
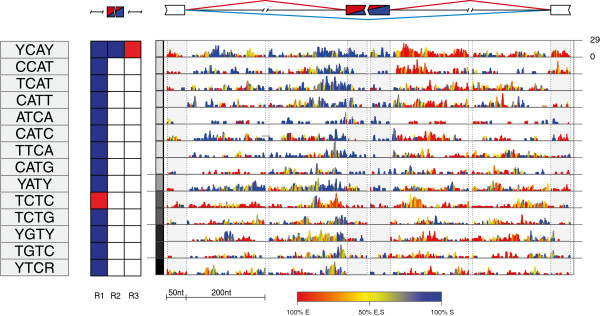
**RNA splicing map of multivalent RNA motifs enriched at NOVA target exons.** Sequences of the enriched tetramers are shown on the left, followed by a color-coded panel showing the regions where tetramer enrichment reached the defined threshold around silenced (blue) or enhanced (red) exons. The gray panel marks the tetramers that overlap in their sequence (marked in the same shade of grey), and the right panel depicts the nucleotide-resolution RNA splicing map of each motif at the enhanced or silenced exons, and their flanking exons. The color key indicates whether the position-specific contribution originates from enhanced (E; red), silenced (S; blue), or both (yellow) sets. The maximum enrichment score (ES) value of the top tetramer, which is used to plot all tetramers, is reported on the right. Nt, nucleotides.

We first analyzed the exons regulated by NOVA to identify the NOVA RNA splicing map. Our approach differed from previous studies [2,25] since we did not predefine the sequence specificity of NOVA, consider motif conservation, or use CLIP data. We analyzed the 98 enhanced, 70 silenced and 4,200 control exons that were identified by the splice junction microarray study of *NOVA2*^*-/-*^ mouse brain neocortex [25] (Additional file 1). Our method identified 14 tetramers enriched at the NOVA-regulated exons (Figure 2; Additional file 2). For the purpose of comparative analysis, tetramers were grouped based on similarity in their sequence. YCAY was the top-ranking tetramer, and 8 of the 14 tetramers enriched at NOVA target exons were part of the YCAY group (Figure 2), in agreement with *in vitro* studies that identified YCAY as the core NOVA-binding motif. All 14 motifs were found significantly enriched upstream of silenced exons, except for TCTC, which was enriched upstream of enhanced exons. YCAY was also enriched within silenced exons and downstream of enhanced exons.

We next assessed how the enriched tetramers are precisely positioned in the RNA splicing map. We determined the enrichment score (ES) at nucleotide resolution and plotted color-coded RNA splicing maps, where blue and red colors represent splicing silencer or enhancer motifs, respectively. The RNA splicing maps showed that the seven tetramers that are part of the YCAY group were enriched at similar positions: downstream of the enhanced exons, and upstream of the silenced exons (Figure 2). These positions were consistent with the map defined originally based on the analysis of conserved YCAY clusters [2]. Importantly, YCAY identified a more complete set of enriched positions compared to the ND tetramers (Figure 2). This result indicates that analysis of DG tetramers can improve the comprehensive identification of splicing regulatory motifs.

### Comparative analysis of RNA splicing maps of diverse RBPs

In order to compare the regulatory properties of multiple RBPs in an unbiased manner, we collected data from multiple studies that have used Affymetrix AltSplice exon junction microarrays to evaluate splicing changes that take place upon knockout or knockdown of an RBP [10,26-28]. RNAmotifs predicted multivalent RNA motifs that agree with the RNA specificity defined for the assessed proteins in the past studies.

**Figure 3:**
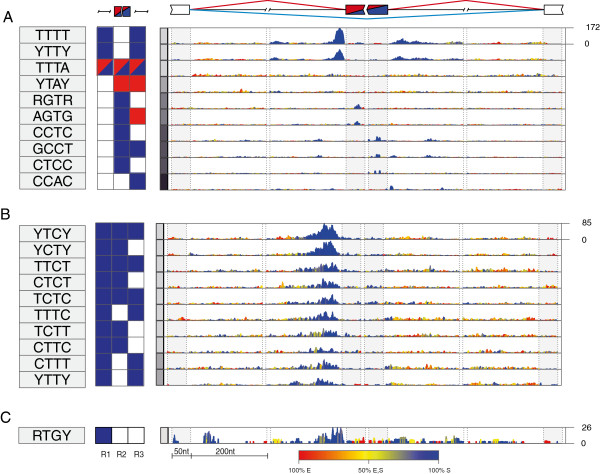
**RNA splicing map of multivalent RNA motifs for RBPs that predominantly repress splicing.** The RNA splicing maps are shown as described in Figure 2 for the following proteins: **(A)** hnRNP C, **(B)** PTBP1, **(C)** TDP-43. The 10 top ranking motifs are shown for hnRNP C and PTBP1. Nt, nucleotides.

**Figure 4:**
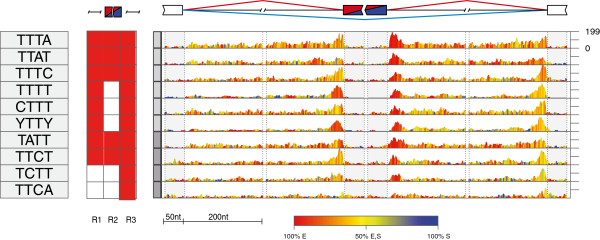
**RNA splicing map of multivalent RNA motifs for TIA1/TIAL1 that predominantly enhance splicing.** The RNA splicing map is shown as described in Figure 2 for TIA1/TIAL1, showing the 10 top ranking motifs. Nt, nucleotides.

The top ranking tetramers predicted for hnRNP C (TTTT and YTTY) were enriched at the 3′ splice sites and in a more widespread region downstream of the silenced exons (Figure 3a), in agreement with the RNA map that was defined based on iCLIP data [26]. Moreover, we identified three additional tetramers with lower enrichment (AGTG, CCTC, CCAC), which most likely correspond to motifs that are common at the Alu-derived exons that are regulated by hnRNP C [29]. The top ranking tetramers predicted for PTB (YTCY and YCTY) were enriched at the 3′ splice sites of the silenced exons (Figure 3b), in agreement with the known PTBP1 binding to TC-rich motifs upstream of the silenced exons [10,30]. A lower enrichment of TC-rich motifs was also observed downstream of enhanced exons (Figure 3b; Additional files 2 and 3), which is consistent with the previous finding that PTBP1 can enhance splicing when binding downstream of alternative exons [10]. The top ranking TARDBP tetramer (RTGY) was enriched at the 3′ splice sites of the silenced exons (Figure 3c), in agreement with the RNA map that was defined based on iCLIP data [27]. Finally, the top ranking TIA tetramers (TTTA, TTAT) were enriched downstream of the enhanced exons (Figure 4), again in agreement with the RNA map that was defined based on iCLIP data [28].

**Figure 5:**
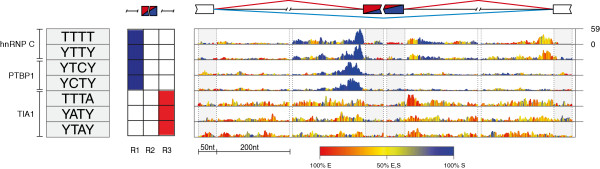
**RNA splicing maps of multivalent RNA motifs enriched in the 'mixed' set of exons regulated by hnRNP C, PTBP1 and TIA.** The RNA splicing map is shown as described in Figure 2 for the mixed data hnRNP C, PTBP1, and TIA1/TIAL1 target exons. Nt, nucleotides.

**Figure 6:**
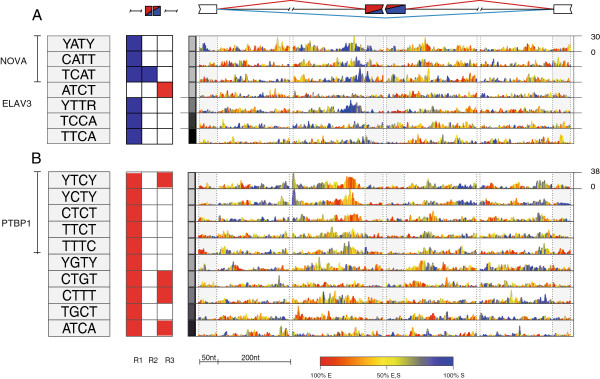
**RNA splicing maps of multivalent RNA motifs enriched at the exons differentially spliced between adult brain and heart.** The RNA splicing maps are shown as described in Figure 2 for a comparison between adult brain and heart splicing. **(A)** Tetramers enriched at positions corresponding to the standard pattern of RNA maps (enrichment in *r*_*1*_ and *r*_*2*_ of exons silenced in the brain, and/or in *r*_*3*_ of exons enhanced in the brain). These tetramers are predicted to be bound by RBPs with higher expression in the brain compared with the heart. **(B)** The 10 top ranking tetramers enriched at positions corresponding to the reciprocal pattern of RNA maps (enrichment in *r*_*1*_ and *r*_*2*_ of exons enhanced in the brain, and/or in *r*_*3*_ of exons silenced in the brain). These tetramers are predicted to be bound by RBPs with higher expression in the heart compared to the brain. Nt, nucleotides.

All of the top ranking motifs are in agreement with the sequence specificity determined by past studies, including the observation that PTBP1 and TARDBP can tolerate pyrimidine or purine transitions in their binding sites [21-23,26,28,30,31]. We also identified other motifs less enriched at exons regulated by these proteins, which will not be discussed here, but could be used for future studies of cooperative splicing regulation (Figures 2, 3, 4, 5 and 6; Additional files 2, 3 and 4). Most importantly, by deriving RNA splicing maps for five distinct RBPs, we could perform an unbiased comparison of their positional splicing effects. NOVA proteins are unique in their ability to repress and enhance exon inclusion to the same extent, since a similar extent of motif enrichment is seen at both types of exons. The RNA splicing map of PTBP1 is most similar to that of NOVA, containing motif enrichment at 3′ splice sites of silenced exons and downstream of enhanced exons, but the enrichment at silenced exons is more dominant in the case of PTBP1. In the case of hnRNP C and TDP-43, the motif enrichment is restricted to the silenced exons, and in the case of TIA, it is largely restricted to the enhanced exons. Nevertheless, the similarity in motif positions suggests that all the assessed proteins repress or enhance exon inclusions from roughly the same positions as NOVA proteins; instead, the differences between the RNA splicing maps of RBPs reflect their variable extent of splicing repression compared with enhancement.

### Co-regulation of alternative exons

YCAY and related tetramers were the most enriched at the exons regulated by NOVA. Nevertheless, three other groups of tetramers were also identified as significantly enriched in NOVA target exons. Of these, the TCTC motif is of particular interest, since it is bound by PTBP1 and PTBP2 (Figure 2). Importantly, this motif was enriched in a reciprocal pattern compared to the PTBP1 RNA map. Rather than being enriched at the 3′ splice sites of silenced exons, it was enriched at the 3′ splice sites of exons that are enhanced by NOVA. To assess the relationship between the YCAY and TCTC motifs, we evaluated the exons showing instances of these tetramers in *r*_*1*_, *r*_*2*_ or *r*_*3*_ regions of interest (see Materials and methods). We identified five exons enhanced by NOVA, which had a YCAY cluster downstream of the 5' splice site (*r*_*3*_) as well as a TCTC cluster upstream of the 3' splice site (*r*_*1*_) (Additional file 5). This number of enhanced exons having both types of motifs in the regions of interest was significantly higher compared to the control exons (*P* = 0.0026, Fisher’s exact test). Since PTBP1 is a potent repressor of neuron-specific splicing [32], it is likely that these exons are silenced by PTBP1 in non-neuronal cells, and enhanced by NOVA in neurons [20], which could increase the fidelity of their brain-specific splicing pattern. This finding demonstrates that RNAmotifs can be used to study co-regulation of alternative exons by RBPs that bind to multivalent regulatory motifs.

### Identification of multivalent motifs mediating tissue-specific alternative splicing

In addition to defining the RNA splicing maps by analysis of exons that change their splicing after removal of a single RBP, we also tested the ability of RNAmotifs to identify regulatory motifs from more complex datasets. Initially, we prepared a dataset where we mixed the alternative exons regulated by hnRNP C, PTBP1 and TIA proteins. We considered exons as differentially expressed if they were in the enhanced or silenced group for any of these proteins, and considered exons as controls if they were regulated by none of these three proteins. Interestingly, in spite of analyzing these mixed data, the eight top-ranking tetramers included the motifs bound by hnRNP C, PTBP1, and TIA proteins (TTTT, YTCY and TTTA, respectively). Furthermore, the three distinct groups of tetramers were correctly separated (TT-rich, TC-rich and TA-rich), and were enriched at the same positions in the RNA splicing maps as in the analysis of unmixed data (Figure 5). This result demonstrates that our algorithm can be applied to studies of complex datasets, which include exons regulated by multiple RBPs.

Tissue-specific patterns of alternative splicing are a result of combinatorial actions of multiple RBPs that are differentially expressed between these tissues. We used Affymetrix AltSplice splice junction microarrays to identify alternative exons that are differentially spliced between adult human brain and heart tissues, and used RNAmotifs to identify regulatory motifs that contribute to the splicing differences. Following the principles of the RNA map, we expected that RBPs that are more highly expressed in the brain will bind to motifs enriched in *r*_*1*_ and *r*_*2*_ of exons silenced in the brain, and/or in *r*_*3*_ of exons enhanced in the brain (see Materials and methods). This identified seven motifs that were enriched upstream of silenced exons and one (ATCT) enriched downstream of enhanced exons (Figure 6a). The top ranking tetrameres were YATY, CATT, TCAT, which all correspond to the YCAY motif bound by NOVA. The other top ranking tetramer was YTTR, which corresponds to the binding motif of ELAV3, a neuron-specific RBP that binds to uridine tracts that are interrupted by a single G or A [33]. Conversely, RBPs that are more highly expressed in the heart would be expected to bind motifs enriched in *r*_*1*_ and *r*_*2*_ of exons enhanced in the brain, and/or in *r*_*3*_ of exons silenced in the brain. We identified 17 tetramers that were enriched upstream of enhanced exons, the top-ranking of which were TC-rich tetramers that were identical to those enriched in the PTBP1 RNA map (Figures 2 and 6b). We could not identify candidate RBPs that recognize the remaining identified motifs; therefore, these could be a basis for future studies. In summary, our predictions agree with past studies showing that NOVA and ELAV3 are expressed in neurons to promote brain-specific splicing, whereas PTBP1 is expressed outside brain to oppose neuron-specific splicing patterns [32]. Thus, in addition to identifying the regulatory motifs, the RNA maps can also predict the tissue where the candidate RBPs binding to these motifs are more highly expressed.

## Discussion

In this study, we have developed and evaluated RNAmotifs software to identify multivalent RNA motifs from analysis of differentially regulated exons. Multivalency plays a pivotal role in biological recognition [34], and its role has been well explored in ubiquitin signaling [35] and in the extracellular matrix [36]. Here we explore the importance of this concept for RNA regulation, and confirm that multivalent RNA motifs mediate the position-dependent splicing regulation. Even though we used no experimental evidence on the sequence specificity of different proteins, RNAmotifs generated RNA splicing maps that were similar to those previously defined by CLIP [10,25-28]. The method does not require analysis of sequence conservation and can therefore identify species-specific regulatory sites. Finally, we used our approach to identify multivalent RNA motifs that mediate tissue-specific splicing regulation. Importantly, the most enriched motifs correspond to binding sites of NOVA, ELAV3 and PTBP1 proteins, and the positions of motif enrichment in the RNA splicing map agree with the higher expression of NOVA and ELAV3 in the brain [20,33], and PTBP1 in other tissues [32].

A comparison of the RNA splicing maps shows that all of the five RBPs silence exon inclusion mainly when binding upstream or within the exons, and enhance inclusion when binding only downstream of exons. However, the RBPs differ in the frequency of their functional binding sites at the different positions of the RNA splicing map. The positions of motif enrichment demonstrate that hnRNP C and TARDBP almost exclusively repress exon inclusion, whereas TIA proteins enhance exon inclusion when binding proximally to the exons. Only the motifs bound by NOVA and PTBP1 are enriched both at enhanced and silenced exons, but in the case of PTBP1, the enrichment is more significant at the repressed exons. Thus, NOVA seems quite unique in its capacity to directly repress or enhance inclusion of a similar number of exons.

Analysis of DG motifs generated a more comprehensive RNA splicing map for NOVA, TDP-43 and PTBP1 compared to ND motifs. This is most clear in the case of NOVA proteins, where only the analysis of YCAY motifs identified the full spectrum of known positions where NOVA proteins regulate splicing (upstream of and within silenced exons, and downstream of enhanced exons). The ND motifs show biases for specific positions; for instance, CCAT clusters are primarily enriched downstream of enhanced exons, and TCAT clusters are enriched upstream of silenced exons. We propose that these positional biases may relate to the other proteins that can bind at the same positions. NOVA may compete with U2AF65 at the 3′ splice sites of silenced exons. U2AF65 preferentially binds to uridine; therefore, enrichment of a uridine-rich TCAT motif at 3′ splice sites may allow U2AF65 to initiate exon inclusion in non-neuronal tissues [37]. In contrast, the exons enhanced by NOVA should not be enhanced by other exons outside the brain, in order to ensure their brain-specific splicing pattern. Therefore, a cytosine-rich CCAT motif downstream of enhanced exons may prevent TIA proteins from binding and initiating exon inclusion in non-neuronal tissues. Thus, the DG binding motif may allow NOVA to adjust the sequence of its binding sites depending on other proteins that can act at the same positions in the RNA splicing map, which could contribute to NOVA’s capacity to either repress or enhance exon inclusion.

Our results support coordinate regulation of exons by multiple RBPs. We identified several exons containing both TCTC and YCAY clusters, indicating reciprocal regulation by PTB and NOVA proteins. The TCTC cluster resembles the binding site of PTBP1 and PTBP2. PTBP1 is a potent repressor of neuron-specific splicing in non-neuronal cells [32]; therefore, the reciprocal regulation of exons by NOVA and PTBP1 could enhance the fidelity of neuron-specific splicing. Moreover, PTBP2 is a neuronal protein that was shown to antagonize the splicing activity of NOVA on *GlyRalpha2* pre-mRNA [14,38]. Since PTBP2 represses adult-specific splicing in neuronal precursors, the reciprocal regulation by NOVA and PTBP2 could ensure that the exons reach their brain-specific pattern in the adult, but not embryonic brain [39].

## Conclusions

RNAmotifs provides a publicly available tool to identify regulatory motifs from analysis of co-regulated alternative exons. We demonstrate how analysis of multivalent RNA motifs and their precise positions can facilitate the prediction of regulatory motifs from splice junction microarray data. RNAmotifs can be readily combined with RNAseq data to assess differentially regulated exons or alternative polyadenylation sites. If combined with additional elements, such as conservation, CLIP binding, RNA structure and analysis of non-clustered contiguous motifs, analysis of multivalent RNA motifs could be further integrated into studies of tissue-specific splicing and the RNA splicing code [7].

## Materials and methods

RNAmotifs software employs the GeCo++ library [40] and the R statistical software [41]. It is freely available via a Bitbucket repository at [42]. The repository includes the processed microarray data that can be used to replicate the results of this study, together with automated scripts that download and prepare the genomic sequence, search for tetramers in splicing regions and compute other analysis steps. Documentation about installing and running the software is available in the README file. The unprocessed cel files of brain and heart splice junction microarray data are available from ArrayExpress with accession number E-MTAB-1911. The microarray data from previous studies that were used here is available from ArrayExpress with accession numbers E-MTAB-527, E-MTAB-526, E-MTAB-367, E-GEOD-12965 and E-GEOD-23513.

### Microarray data

The present study analyzed regulatory motifs at exons identified by Affymetrix AltSplice exon junction microarray experiments from this study and previously published studies. These include analysis of *NOVA1/NOVA2* knockout mouse brain, and knockdown of *hnRNPC*, *PTBP1*, *TPD-43* or *TIA* in human cell lines [10,25-28]. The total adult human brain or adult human heart RNAs were obtained from BioChain and evaluated in triplicates using the same procedure as described previously [27]. The microarray data were analyzed using ASPIRE version 3 [26]. By analyzing the signal of reciprocal probe sets, ASPIRE3 was able to monitor splicing of 18,531 and 9,103 alternative cassette exons (CEs) for human and mouse arrays, respectively. Then, for each RBP, we divided CEs into three sets according to the dIRank: enhanced (dIRank >1), silenced (dIRank < -1) and control exons (| dIRank | <0.1) (Additional file 1). Exon coordinates were retrieved from the UCSC annotation database [43], using the mm9 assembly for mouse and the hg19 assembly for the human exons.

### Definition of multivalent motifs

To define and identify the multivalent motifs, we evaluated three regions around the alternative CEs, which were selected based on the past studies of the Nova RNA splicing map [2]: first, region *r*_*1*_ [-35:-5] nucleotides of intronic sequence upstream of the 3′ splice site; second, region *r*_*2*_ of exonic sequence [1:31] nucleotides downstream of the 3′ splice site and [-31:-1] nucleotides upstream of the 5′ splice site (if exon is shorter than 61 nucleotides, then evaluate the whole exon); third, region *r3* [10:40] nucleotides of intronic sequence downstream of the 5′ splice site (Figure 1).

Since most RNA-binding motifs recognize up to four nucleotides [19], we considered tetramers as our core motifs. Several RBPs tolerate purine or pyrimidine transitions at some positions of their target motifs [17,22,44] so we included 64 DG tetramers in addition to 256 ND tetramers. The 64 DG tetramers were defined such that the central two nucleotides were ND (A,C,G,T), whereas the nucleotides at the boundary could be either purines R(A/G) or pyrimidines Y(C/T), such as in the YCAY tetramer. Thus, each DG tetramer included four ND tetramers. For example, YCAY included instances of TCAT, TCAC, CCAT and CCAC tetramers. We allowed overlap between tetramers when identifying their positions in the sequence (see example below).

We first identified all nucleotides that overlapped with each tetramer, which we refer to as 'tetramer nucleotides'. As the aim of our analysis was to account for the ability of RBPs to bind multiple proximal motifs (that is, multivalent motifs), we determined the 'cluster height' (*h*) as the number of 'tetramer nucleotides' within a 31 nucleotide window centered on each evaluated position; *h* was assigned only to positions directly overlapping a tetramer (see the example below). Hence, *h* ranged from a minimum of 4 to a maximum of 31 (4 ≤ *h* ≤ 31). We analyzed the *h* value for all tetramers at each nucleotide in the sequence surrounding all monitored CEs and their flanking exons.

We retrieved 500 nucleotides of flanking intronic sequence next to each splice site (or up to the middle if the intron is shorter than 1 kb), and 100 nucleotides of exonic sequence next to each splice site (or up to the middle if the exon is shorter than 200 nucleotides) and determined the percentage of genomic sequence (named 'coverage percentage') covered by each tetramer. For each tetramer, we then selected the minimum *h* (*h*_*min*_) corresponding to the coverage percentage closest to 0.5% and then considered all nucleotide positions with *h* ≥ *h*_*min*_ as having the 'cluster instance'. This definition of minimum *h* was made in order to take into account the variation in the occurrence and clustering of different tetramers. To determine the optimal *h*_*min*_, we analyzed the sequences in the regions *r*_*1*_, *r*_2_ and *r*_3_ surrounding the NOVA-regulated enhanced, silenced and control exons, and determined the coverage percentage covered by each *h* for the YCAY tetramer. We assessed the *h* with coverage percentages closest to 0.062, 0.125, 0.25, 0.5, and 1, and the results of this analysis are shown in Additional file 6. A coverage percentage of 0.5% is the minimum required to detect >10% of the regulated exons in each of the regions in a correct manner: that is, silenced exons in regions 1 and 2, and enhanced exons in region 3. After identifying the *h*_*min*_ with a coverage percentage closest to 0.5% for each tetramer, we considered all nucleotide positions with *h* ≥ *h*_*min*_ as having the 'cluster instance'. In this way, we ensured that the probability of a cluster instance with the chosen *h*_*min*_ was similar for all tetramers.

The following example shows the search for the YCAY motif cluster with an *h*_*min*_ of 9 for an arbitrary sequence:

The first line shows the genomic sequence, the second line marks the positions overlapping YCAY tetramers, the third line shows the *h* values, and the last line the positions of the cluster instance. We repeated the analysis for all selected exons and collected the cluster instances of all tetramers.

### Identification of enriched multivalent motifs

To identify the multivalent motifs that occurred in a specific region more often in the regulated exons compared with control exons, we used the following procedure. If any nucleotide sequence included a tetramer with a cluster instance 1 within the region, then the region was given the value of 1, otherwise 0. We then calculated the significance of tetramer enrichment in each of these regions at all enhanced or silenced exons, compared with controls. Formally, let *T* = {*t*_*1*_, *t*_*2*_, *…*, *t*_*320*_} represent tetramers to be analyzed, and *R* = {*r*_*1*_, *r*_*2*_, *r*_*3*_} be the set of regions of interest. For each group of exons (enhanced, silenced and controls), we generated a regional-specific occurrences matrix *M = {R*x*T}*, with three rows and *T* columns. Each cell *M(r*,*t)* represents the sum of values for all exons of the corresponding region *r*_*i*_ and tetramer *t*_*j*_. To evaluate the statistical significance of the region-specific enrichment of each tetramer, we used a one-tailed Fisher’s exact test to test the null hypothesis that the number of cluster instances at a region *r*_*i*_ of a tetramer *t*_*j*_ is not different between enhanced (or silenced) and control exons. A hypothetical example reported in Table 1 shows the test made for a specific region *r*_*i*_ and tetramer *t*_*j*_, assuming that the sum of values is 30 for a total of 98 enhanced exons and 300 for a total of 4,200 control exons. The resulting *P*-value for this example is 6.695 × 10^-12^.

**Table 1:** An example of the values used for the Fisher's exact test of tetramer enrichment

***t***_***j***_		***M***_***enhanced***_ ***(r***_***i***_ ***,t***_***j***_ ***)***	***M***_***control***_ ***(r***_***i***_ ***,t***_***j***_ ***)***	**Total**
Number of exons with tetramer *t*_*j*_	Present	30	300	330
	Absent	98-30	4,200-300	4,298-330
	Total	98	4,200	4,298

The result of this analysis was two matrices *F* (enhanced and silenced, respectively) with three rows and *T* columns of *P*-values representing tetramer enrichments in each region. These *P*-values were corrected for multiple testing relative to the number of tested tetramers (320 in this case) using the Benjamini-Hochberg false discovery rate correction to obtain p_*fdr*_.

We next calculated the achieved significance level of the Fisher’s exact test using a bootstrap procedure (p_empirical_), representing the probability of observing at least that large a value when the null hypothesis is true. p_empirical_ was calculated from 10,000 bootstrap samples of our data. Bootstrap samples were generated by random selection with replacement of the enhanced, silenced and control exons. For each bootstrap sample, the same procedure as described above (including false discovery rate correction) was used to estimate the statistical significance of region-specific tetramer enrichment (p_bootstrap_). The achieved significance of tetramer enrichment was estimated by:

$$p_{\mathrm{empirical}}\left(r_{i},t_{j}\right)=\frac{1+\#\left(p_{\mathrm{bootstrap}}\left(r_{i},t_{j}\right)<p_{\mathrm{fdr}}\left(r_{i},t_{j}\right)\right)}{1+\#\mathrm{bootstrap}}$$

For subsequent analyses we retained tetramers that passed the threshold p_*fdr*_ ≤ 0.1 and p_empirical_ ≤ 0.0005 in any of the three regions, as described below:

$$\begin{array}{l} \left[\left(p_{\mathrm{fdr}}\left(r_{1},t_{j}\right)\leq 0.1\;\mathrm{AND}\;p_{\mathrm{empirical}}\left(r_{1},t_{j}\right)\leq 0.0005\right)\right]\;\mathrm{OR}\;\left[\left(p_{\mathrm{fdr}}\left(r_{2},t_{j}\right)\leq 0.1\;\mathrm{AND}\;p_{\mathrm{empirical}}\left(r_{2},t_{j}\right)\leq 0.0005\right)\right]\\ \mathrm{OR}\;\left[\left(p_{\mathrm{fdr}}\left(r_{3},t_{j}\right)\leq 0.1\;\mathrm{AND}\;p_{\mathrm{empirical}}\left(r_{1},t_{j}\right)\leq 0.0005\right)\right] \end{array}$$

We evaluated tetramer enrichment in the enhanced and silenced set independently of each other. The tetramers that passed the threshold are reported in Additional files 2, 3 and 4. These tetramers were studied with the further steps, ending with the RNA map visualization.

### Nucleotide-resolution RNA maps of motif enrichment

To visualize the precise positions in the pre-mRNA where clusters are enriched, we performed a position-specific enrichment analysis at positions corresponding to the exon-intron and intron-exon junctions of alternative CEs and flanking exons extending 200 nucleotides into introns and 50 nucleotides into exons. If the intron or exon were shorter than 400 or 100 nucleotides, respectively, we evaluated the sequence as far as the middle of the intron or exon. In these regions, we determined the positions of cluster instances for all tetramers. Formally, let *T* = {t_1_, t_2_, …, t_320_} represent tetramers to be analyzed and let *P* = {p_1_, p_2_, …, p_1000_} be the set of positions of interest (250 nucleotides for each of the four considered junctions). For each group of exons (enhanced, silenced and controls), we generated a positional-specific occurrences matrix *M = {P*x*T}*, with *P* rows and *T* columns. Each cell *M(p*,*t)* represents the number of cluster instances at position *p*_*i*_ of the tetramer *t*_*j*_. To evaluate statistical significance of position-specific enrichment of each tetramer, we used a Fisher’s exact test to test the null hypothesis that the number of cluster instances at a position *p*_*i*_ of a tetramer *t*_*j*_ is not different between enhanced (or silenced) and control exons. The result of this analysis was two matrices *F* (enhanced and silenced, respectively) with *P* rows and *T* columns of position-specific *P*-values representing tetramer enrichments.

We next evaluated the position-specific occurrences of each tetramer at two distinct sets of exons (that is, enhanced and silenced exons). We used the Fisher’s method [45] to combine the two independent tests into one goodness-of-fit (Χ^2^) statistic, referred to as the enrichment score (ES). The ES of each selected tetramer at each position in the regions of interest was calculated using the following formula:

$$\mathrm{ES}\left(p,j\right)=-2*\left[\log\left(F{\left(p,j\right)}_{\mathrm{Enchanced}}\right)+\log\left(F{\left(p,j\right)}_{\mathrm{Silenced}}\right)\right]$$

with {*p* ∈ *P*} (positions) and {*j* ∈ *T*: *p*_*empirical*_ ≤ *α*} (selected tetramers).

For alternative exons, ES allows evaluation of the joint enrichment at enhanced and silenced exons. To visualize the splicing regulatory activity of each tetramer at enhanced and silenced exons separately, we then used the RNA splicing maps as described below.

Tetramers were grouped on the basis of sequence composition and ES profile. For each tetramer we calculated the cumulative sum of ES over the positions. We next aligned the remaining tetramers to the one with the highest cumulative sum, and whenever the alignment of another tetramer matched three consecutive nucleotides, it was grouped together with the top tetramer. We recursively repeated the procedure on non-aligned tetramers until all were part of 'groups'. In the case of DG motifs, the alignment of each motif was performed using the four ND sequences composing the motif and requiring at least two ND sequences to be aligned. Within each group, tetramers were sorted on the basis of the Pearson’s correlation of their enrichment profile with the top scored tetramers of the group.

We visualized the RNA splicing maps by plotting the ES profiles over the region of interest (Figure 1). All RNA maps display the enrichment score normalized to the maximum value in the ES matrix. In cases where more than 10 tetramers were retained with the p_*fdr*_ ≤0.1 and p_empirical_ ≤0.0005 threshold (Additional file 4), the RNA maps in Figures 3, 4, 5 and 6 show only the 10 tetramers with the highest maximum ES values. The color key indicates the contribution of enhanced (red = 100%), silenced (blue = 100%) or both (yellow = 50%) sets of exons to the position-specific enrichment of a tetramer. Thus, the RNA map does not exclude examples where both enhanced and silenced exons are enriched at the same position; whereas red and blue show motifs enriched only at enhanced or silenced exons, positions where motifs are enriched in both sets of exons are shown in yellow.

## Abbreviations

CE: cassette exon; CLIP: crosslinking and immunoprecipitation; DG: degenerate; ES: enrichment score; ND: non-degenerate; RBP: RNA-binding protein.

## Competing interests

The authors declare that they have no competing interests.

## Authors’ contributions

MC and JU conceived the project and wrote the paper; MC and GR developed the software and performed the analysis, with assistance from PJ; AS and TC generated microarray data from brain and heart, UP and JU supervised the study. All authors read and approved the final manuscript.

## Supplementary Material

Additional file 2 **Tetramers found enriched in at least one dataset.** This figure shows tetramers that have been found significantly enriched (*p*_*dfr*_ <0.1 and *p*_*empirical*_ <0.0005) in at least one dataset (NOVA, hnRNP C, PTBP1, TARDBP, TIAL1, 'Mixed' and Brain-Heart sets) in the region of interest surrounding the cassette exons. Tetramers enriched in enhanced exons are depicted in blue, whereas those enriched in silenced exons are in red. Click here for file

Additional file 1 **Evaluation of the NOVA target exons identified when different 'coverage percentages' are chosen for the definition of YCAY clusters.** This figure shows the analysis that was used to determine the appropriate *h*_*min*_. We retrieved 500 nucleotides of flanking intronic sequence next to each splice site (or up to the middle if the intron is shorter than 1 kb), and 100 nucleotides of exonic sequence next to each splice site (or up to the middle if the exon is shorter than 200 nucleotides) and determine the percentage of genomic sequence (termed 'coverage percentage') covered by each tetramer. To determine the optimal *h*_*min*_, we analyzed the sequences in the regions *r*_*1*_, *r*_2_ and *r*_3_, and determined the coverage percentage for each *h* for the YCAY tetramer. The percentage of NOVA-regulated enhanced, silenced and control exons that contained the YCAY tetramer differed when choosing the coverage percentage closest to 0.062, 0.125, 0.25, 0.5, and 1, as shown separately for each region in the three graphs. As shown in the table, 0.5% is the minimum coverage percentage required to detect >10% of the regulated exons in each of the regions in a correct manner: that is, silenced exons in regions 1 and 2, and enhanced exons in region 3. Click here for file

Additional file 3 **Table showing the number of regulated and control cassette exons for each RNA-binding protein.** The 'Mixed' dataset contains exons that are differentially regulated by hnRNP C, PTB or TIA proteins. For this set, we considered only exons showing the same regulatory activity with the three proteins. No overlap between enhanced, silenced and control exons was allowed. (XLS 405 kb) Click here for file

Additional file 4 **Table showing results of enrichment analysis of tetramer clusters at exons regulated by different RBPs.** Each line shows the tetramer, its *p*_*fdr*_, and *p*_*empirical*_ obtained from 10,000 bootstrap samples for the three region of interest. Each sheets reports data for a specific data set (NOVA, hnRNP C, PTBP1, TARDBP, TIAL1, 'Mixed' and Brain-Heart sets). Click here for file

Additional file 5 **Table showing tetramers found as significantly enriched.** Each line reports an enriched tetramer for an exon type (enhanced, silenced), the regions where it was found as significantly enriched, the corresponding *p*_*fdr*_, and *p*_*empirical*_ obtained from 10,000 bootstrap samples for the three regions of interest in a specific data set (NOVA, hnRNP C, PTBP1, TARDBP, TIAL1, 'Mixed' and Brain-Heart sets). Click here for file

Additional file 6 **Table showing Nova-targeted exons co-regulated by PTBP1.** The table reports exons that show instances of both YCAY and TCTC clusters. Click here for file
