# Supplementary material for: RNAmotifs: prediction of multivalent RNA motifs that control alternative splicing
Source: Genome Biol. 2014 Jan 31;15(1):R20. doi: 10.1186/gb-2014-15-1-r20 (PMC4054596; doi:10.1186/gb-2014-15-1-r20)
Supplement: Additional file 4 — Table showing results of enrichment analysis of tetramer clusters at exons regulated by different RBPs. Each line shows the tetramer, its pfdr, and pempirical obtained from 10,000 bootstrap samples for the three region of interest. Each sheets reports data for a specific data set (NOVA, hnRNP C, PTBP1, TARDBP, TIAL1, 'Mixed' and Brain-Heart sets). [file gb-2014-15-1-r20-S4.pdf]

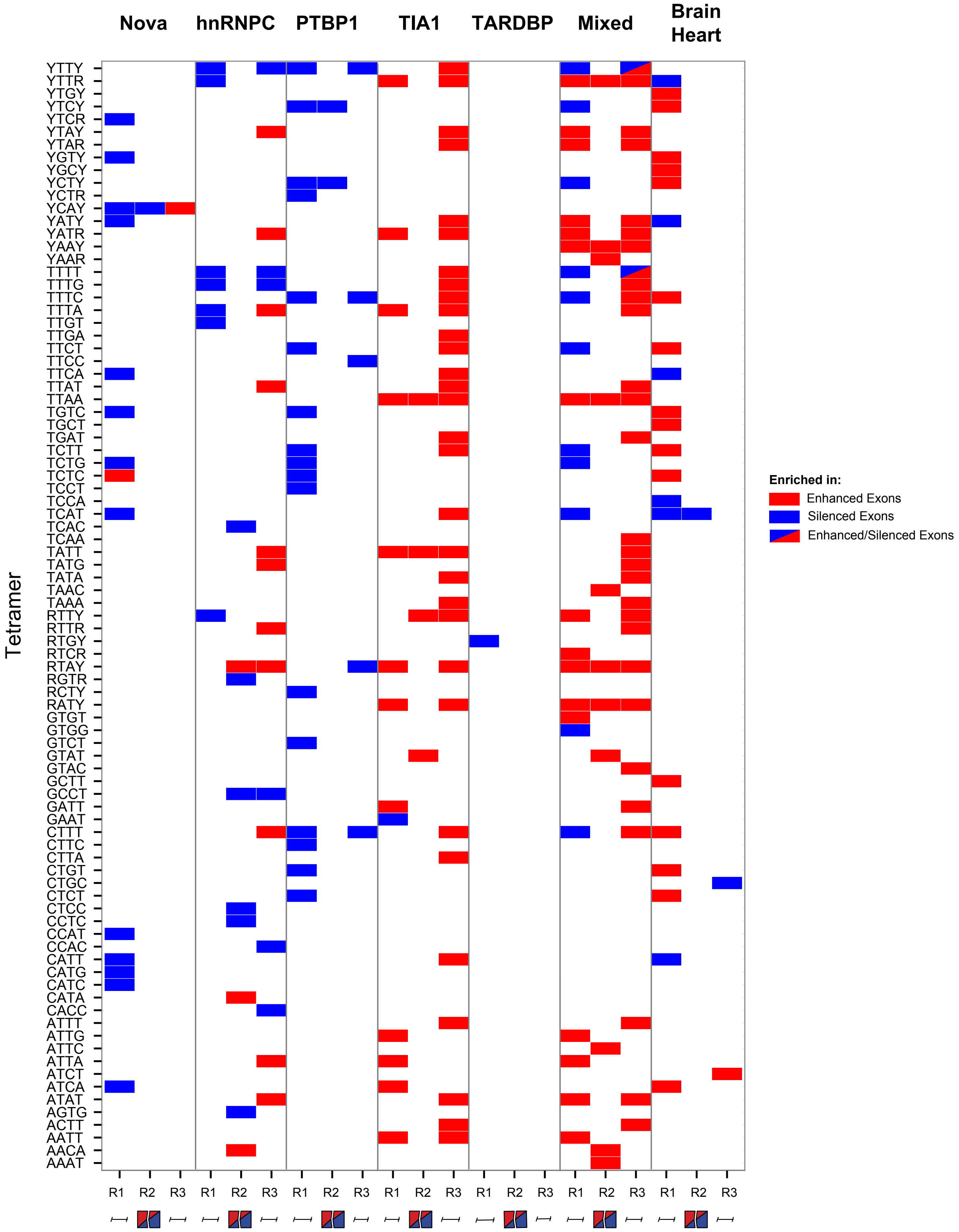

Nova
hnRNPC
PTBP1
TIA1
TARDBP
Mixed
Brain Heart
Tetramer
YTTY
YTTR
YTGY
YTCY
YTCR
YTAY
YTAR
YGTY
YGCY
YCTY
YCTR
YCAY
YATY
YATR
YAAY
YAAR
TTTT
TTTG
TTTC
TTTA
TTGT
TTGA
TTCT
TTCC
TTCA
TTAT
TTAA
TGTC
TGCT
TGAT
TCTT
TCTG
TCTC
TCCT
TCCA
TCAT
TCAC
TCAA
TATT
TATG
TATA
TAAC
TAAA
RTTY
RTTR
RTGY
RTCR
RTAY
RGTR
RCTY
RATY
GTGT
GTGG
GTCT
GTAT
GTAC
GCTT
GCCT
GATT
GAAT
CTTT
CTTC
CTTA
CTGT
CTGC
CTCT
CTCC
CCTC
CCAT
CCAC
CATT
CATG
CATC
CATA
CACC
ATTT
ATTG
ATTC
ATTA
ATCT
ATCA
ATAT
AGTG
ACTT
AATT
AACA
AAAT
R1 R2 R3 R1 R2 R3 R1 R2 R3 R1 R2 R3 R1 R2 R3 R1 R2 R3 R1 R2 R3
Enriched in:
Enhanced Exons
Silenced Exons
Enhanced/Silenced Exons
