# Supplementary material for: RNAmotifs: prediction of multivalent RNA motifs that control alternative splicing
Source: Genome Biol. 2014 Jan 31;15(1):R20. doi: 10.1186/gb-2014-15-1-r20 (PMC4054596; doi:10.1186/gb-2014-15-1-r20)
Supplement: Additional file 6 — Table showing Nova-targeted exons co-regulated by PTBP1. The table reports exons that show instances of both YCAY and TCTC clusters. [file gb-2014-15-1-r20-S6.pdf]

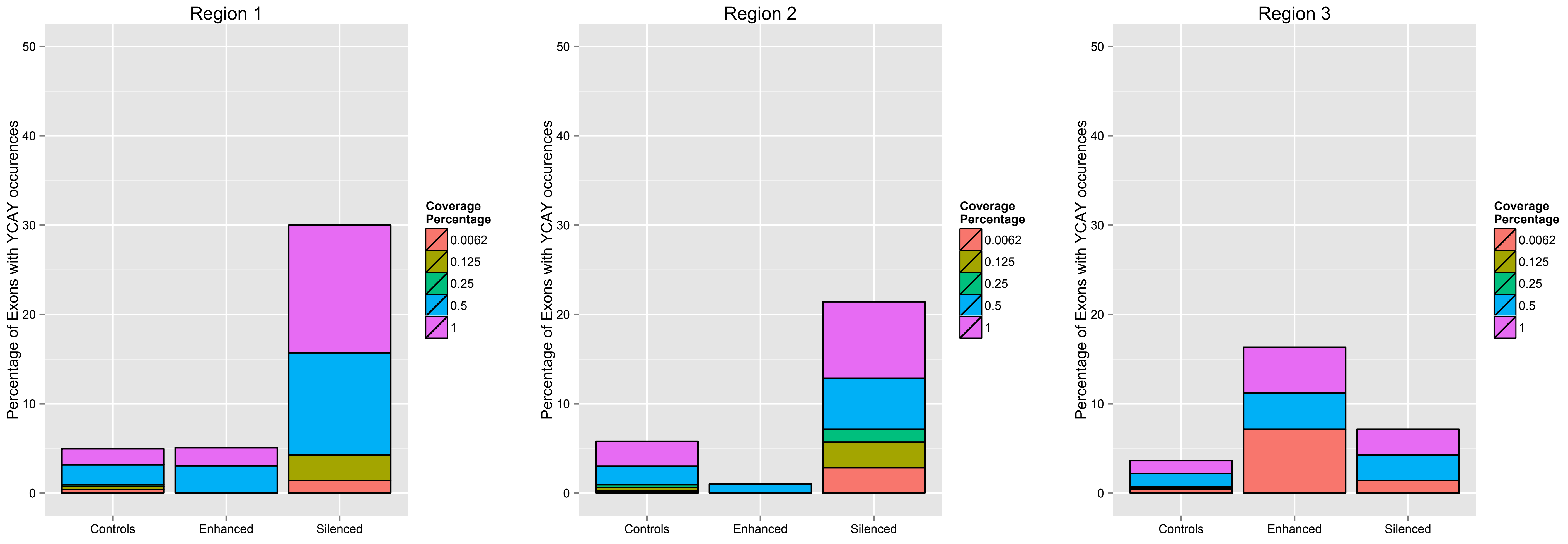

Coverage Percentage	Exon type	Number of Exon with YCAY occurences in:			Percentage of Exon with YCAY occurences in:		
		region 1	region 2	region 3	region 1	region 2	region 3
1%	Enhanced	5	1	16	5%	1%	16%
	Silenced	21	15	5	30%	21%	7%
	Controls	209	243	153	5%	6%	4%
0.5%	Enhanced	3	1	11	3%	1%	11%
	Silenced	11	9	3	16%	13%	4%
	Controls	134	127	92	3%	3%	2%
0.25%	Enhanced	0	0	7	0%	0%	7%
	Silenced	3	5	1	4%	7%	1%
	Controls	40	40	29	1%	1%	1%
0.125%	Enhanced	0	0	7	0%	0%	7%
	Silenced	3	4	1	4%	6%	1%
	Controls	32	27	25	1%	1%	1%
0.0062%	Enhanced	0	0	7	0%	0%	7%
	Silenced	1	2	1	1%	3%	1%
	Controls	17	11	20	0%	0%	0%
